# Offset reconstruction in stemless and stemmed shoulder hemiarthroplasty: influence on long-term outcomes

**DOI:** 10.1007/s00402-026-06314-3

**Published:** 2026-06-03

**Authors:** Raphael Trefzer, Amina Gurda, Julian Deisenhofer, Johannes Weishorn, Mustafa Hariri, Kevin-Arno Koch, Matthias Wolf, Matthias Bülhoff

**Affiliations:** Department of Orthopaedics, Heidelberg University, Heidelberg, Germany

**Keywords:** Shoulder hemiarthroplasty, Long-term outcomes, Offset, Joint reconstruction

## Abstract

**Background:**

Shoulder hemiarthroplasty (HA) is a well-established treatment for various humeral-side pathologies. Overstuffing should be avoided because it is associated with glenoid wear; however, specific radiological reconstruction targets remain unclear. This study assessed the association between radiological joint restoration in stemmed and stemless HA and long-term functional outcomes.

**Methods:**

Patients who underwent stemmed or stemless HA between 2001 and 2011 were included in a long-term follow-up. Cases with incomplete or non-calibratable digital radiographs or concomitant glenoid procedures were excluded. Clinical outcomes were evaluated using the Constant-Murley Score (CMS) and satisfaction questionnaires. Radiographic parameters—lateral glenohumeral offset (LGHO), lateral humeral offset (LHO), and center of rotation (COR)—were measured on standardized anteroposterior radiographs pre- and postoperatively by two independent observers. Differences from baseline to final follow-up (ΔLHO, ΔLGHO, ΔCOR) were calculated. Interobserver reliability was determined using the intraclass correlation coefficient (ICC). Associations between radiological measures and clinical outcomes were analyzed using linear regression; intergroup comparisons used unpaired t-tests (*p* < 0.05).

**Results:**

Forty-eight patients (12 stemmed, 36 stemless HA) were examined after a mean follow-up of 16.6 ± 2.5 years. CMS did not differ between groups (stemmed 50.5 ± 20.8 vs. stemless 54.1 ± 20.4, *p* = 0.603). Complete radiographic data were available for 25 patients. Interobserver agreement was excellent for LHO (ICC = 0.93) and very good for LGHO (ICC = 0.85). Mean reconstruction values were close to zero for LHO (ΔLHO = − 0.3 ± 5.1 mm), slightly underreconstructed for LGHO (ΔLGHO = − 6.3 ± 12.9 mm), and slightly medialized for COR (ΔCOR = − 3.5 ± 8.5 mm). Linear regression revealed a significant negative association between ΔLHO and CMS (R² = 0.45; coefficient = − 4.9; *p* = 0.02).

**Conclusion:**

Both stemmed and stemless HA achieve satisfactory long-term outcomes after over 16 years. Accurate reconstruction of the lateral humeral offset correlates with improved functional results.

**Supplementary Information:**

The online version contains supplementary material available at 10.1007/s00402-026-06314-3.

## Introduction

Shoulder hemiarthroplasty (HA) is a well-established treatment option for various humeral head pathologies, including primary and post-traumatic osteoarthritis as well as avascular necrosis, particularly when the glenoid remains relatively preserved [2, 9, 12, 16, 17, 27]. As total shoulder arthroplasty (TSA) is associated with significant long-term failure rates – primarily due to glenoid wear and loosening of the glenoid component – HA can still represent a viable implant choice, especially for younger or more active patients [2, 17, 20, 23, 24]. However, long-term outcomes after HA can vary, and replicating native biomechanics remains surgically challenging [11].

Inaccurate anatomical restoration—particularly humeral overstuffing—is associated with complications such as pain, stiffness, rotator cuff dysfunction, and progressive glenoid erosion [10, 14, 21].

Long-term comparative data between stemmed and stemless HA remain limited, and the specific influence of radiographic reconstruction parameters on patient-reported outcomes over extended follow-up periods is sparsely studied.

This study aims to investigate the association between joint reconstruction parameters and long-term clinical outcomes in patients who underwent shoulder HA with stemmed and stemless implants. We hypothesized that closer restoration of the respective reconstruction parameters is associated with better long-term functional outcomes, independent of implant type.

## Methods

### Patients

In this monocenter study, patients who underwent stemmed or stemless HA documented in a local prospective registry database between January 2001 and November 2011 and completed a long-term clinical and radiological follow-up were included. Patients with incomplete digital radiographs or non-calibratable images and patients who received concomitant glenoid interventions were excluded. Indications included primary osteoarthritis, posttraumatic osteoarthritis and humeral head necrosis.

The initial cohort comprised 108 patients who underwent stemless HA and 61 patients with stemmed HA in our database. Indications included primary and posttraumatic osteoarthritis and humeral head necrosis. Of this cohort, 21 patients were deceased, 34 patients could only be reached via telephone and gave information about their prosthesis status but refused or were unable to come for a follow-up examination, 27 patients underwent revision, 39 were lost to follow-up, and a total of 48 patients completed the long-term clinical follow-up examination and were therefore eligible for analysis. As preoperative images were comparably old, complete radiographic documentation was available for 25 patients; the other 23 had non-comparable preoperative radiographs that were either unavailable in digital form or not calibrated for measuring. The baseline demographics of the 25 patients with complete radiological follow-up vs. the 23 patients with incomplete radiological follow-up did not differ (Supplemental Table 1).

All patients signed informed consent preoperatively upon entry in the database and at the follow-up assessment. Ethical approval was obtained by the local ethical committee (S305-2007). The study was conducted in accordance with the Helsinki Declaration of 1975, as revised in 2013. Informed consent was obtained from all participating patients.

### Surgical technique and implants

The cemented Aequalis system (Tornier/Stryker, Michigan, USA) was used in all patients undergoing stemmed shoulder HA. For stemless shoulder HA, the Copeland Shoulder (Biomet Europe, Dordrecht, The Netherlands), Epoca RH Cup (Argomedical, Cham, Switzerland), or Simpliciti System (Tornier/Stryker, Michigan, USA) were implanted. All procedures were performed in beach chair position through a deltopectoral approach with detachment of the subscapularis tendon and capsular release. The humeral neck cut was performed anatomically at the level of the anatomic neck with the aim of restoring native humeral inclination and version. Head sizing and positioning were based on intraoperative assessment of the resected head and anatomical landmarks to reconstruct humeral offset and joint line height. Trial components were used to optimize soft-tissue tension and joint stability before definitive implantation.

Primary stability of the implant and restoration of joint mechanics were assessed manually. The subscapularis tendon was repaired using tendon-to-tendon sutures.

### Clinical evaluation and patient reported outcomes (PROs)

Functional and clinical outcomes were assessed using the Constant-Murley Score (CMS) [6], which served as the primary endpoint. Isometric abduction strength was assessed using an Isobex dynamometer (Cursor AG, Bern, Switzerland) with the patient standing and the respective shoulder at 90 degrees of abduction.

Subjective evaluation was conducted using the Simple Shoulder Test (SST), which consists of patient-reported responses regarding shoulder mobility and function in daily activities. In addition, the Subjective Shoulder Value (SSV) was assessed.

### Radiological evaluation

**Fig. 1:**
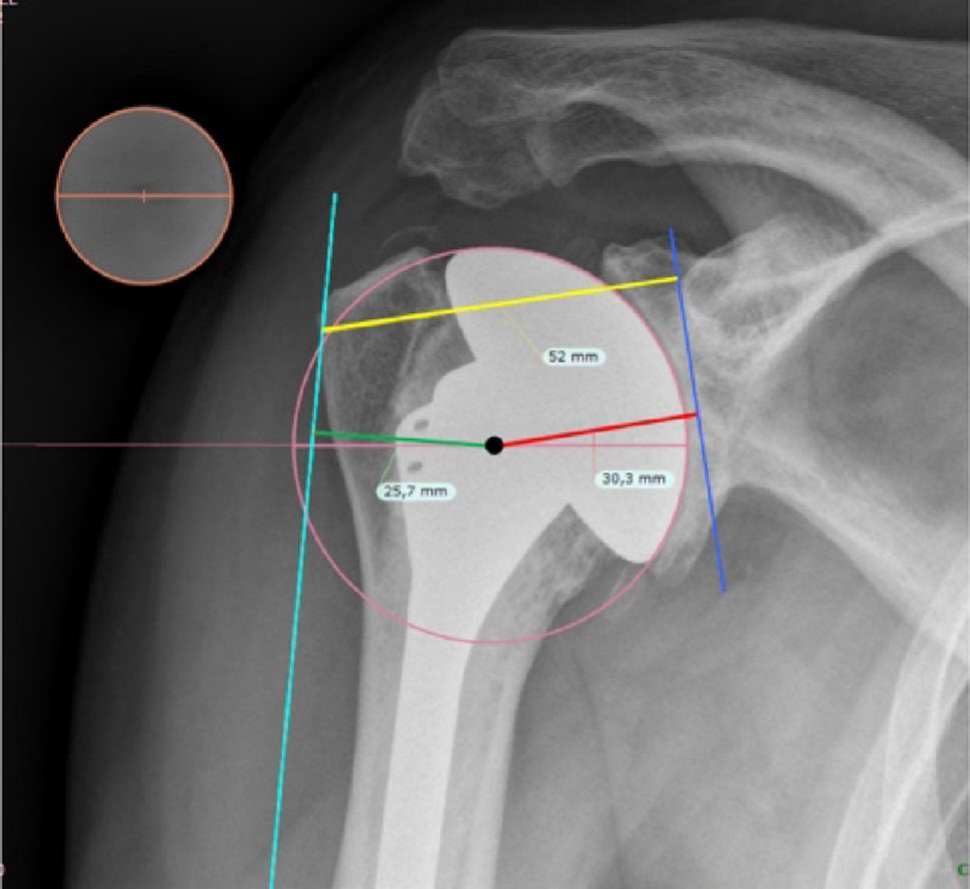
Offset measurement method: The orange circle represents the calibration ball with a diameter of 25.4 mm. The Center of Rotation (COR) is determined as the center of the circle radius of the prosthesis or humeral joint surface. The blue reference line tangential to the coracoid base and parallel to the glenoid surface serves as the scapular reference for the offset measurements. The yellow Lateral Glenohumeral Offset (LGHO) line is drawn from the greater tubercle (GT) perpendicular to the blue reference line, making it the shortest distance between the two points. Lateral Humeral Offset (LHO) is measured as the shortest distance from the GT to the COR and is represented by the green line: From the COR perpendicular to a humeral reference line (turquoise) tangential to the lateral edge of the GT and parallel to the humerus shaft. COR offset is measured with the red line from the COR perpendicular to the blue scapular reference line

Radiographic measurements were taken by two independent observers (RT and AG) on standard true anteroposterior of the shoulder obtained during routine clinical care preoperatively and at the follow-up examinations in one measurement procedure, blinded to clinical outcomes, using the TraumaCad software (Brainlab, Munich, Germany). For analysis of joint reconstruction parameters, measurements were taken as follows (Fig. 1): Lateral glenohumeral offset (LGHO), defined as the distance from the lateral edge of the greater tuberosity (GT) to the base of the coracoid process; and lateral humeral offset (LHO), defined as the distance from the lateral edge of the GT to the center of rotation (COR), as previously described [19]. COR offset was calculated as LGHO subtracted by LHO, defining the distance between the COR to the base of the coracoid process, serving as the constant landmark. The differences of each variable from preoperatively to the latest follow-up were calculated for each patient: ΔLHO, ΔLGHO and ΔCOR.

### Statistical evaluation

Statistical analyses were performed using SPSS Version 26.0 (IBM^®^, Armonk, New York, USA). Preoperative data were retrieved from the local registry database and were tabulated according to the cases included in the study.

Intraclass correlation coefficients (ICC) were determined to evaluate inter-observer reliability. Linear regression was used to assess the association of radiological reconstruction measures with clinical outcome. For the correlation analysis of clinical outcomes to the postoperative deviation from preoperative offset parameters, the absolute numbers of ΔLHO, ΔLGHO and ΔCOR were used in the regression. As the regression analysis only included patients with complete paired radiographic measurements preoperatively and at latest follow-up, the effective sample size was determined by data availability. A multivariable linear regression model was constructed including abs. ΔCOR offset, abs. ΔLHO and abs. ΔLGHO without automated variable selection. Model fit was assessed using R² and adjusted R², and regression coefficients are reported with 95% confidence intervals.

A post-hoc power analysis for the overall regression model (fixed model, R² deviation from zero) was performed. Based on the observed R² = 0.46, total sample size *n* = 25, and three predictors (α = 0.05), the corresponding effect size was Cohen’s f² = 0.85, yielding an observed power of approximately 0.95 for the global F-test. Normality of residuals was assessed using Q–Q plots and the Shapiro–Wilk test (*p* = 0.19), homoscedasticity using the Breusch–Pagan test (*p* = 0.17), and multicollinearity using variance inflation factors. Missing data were handled using a complete-case approach, as offset deviations required paired preoperative and follow-up radiographs.

Unpaired t-tests were used for intergroup comparison. Significance level was set to *p* < 0.05.

## Results

### Clinical results

A total of 48 patients completed the clinical follow-up examination at a mean of 16.6 years. There were 26 (54%) women and 22 (46%) men. Indications for HA were primary glenohumeral osteoarthritis (OA) in 21 cases, humeral head necrosis in 14 cases, posttraumatic OA in 8 cases, OA with rotator cuff defects in 4 cases and humeral-side joint destruction after tumor extirpation of the humeral head in one case. The cohort included 12 patients with stemmed HA and 36 patients with stemless HA (Table 1). Long-term clinical outcomes were similar across stemmed and stemless implants, range of motion tended to be higher in the stemless group, although differences were not statistically significant (Table 2).

**Table 1:** Baseline demographics

Parameter	Stemmed (Mean ± SD)	Stemless (Mean ± SD)	Difference (Mean, 95% CI)	*p*-value (significance: <0.05)
Patient number	12	36		
Age	55.3 ± 9.6	55.0 ± 10.7	-0.3 (-7.2 to 6.6)	*0.924*
Follow-up [months]	204.7 ± 28.5	197.8 ± 30.3	-6.9 (-27.0 to 13.2)	*0.496*

**Table 2:** Clinical outcomes

Outcome	Stemmed (Mean ± SD)	Stemless (Mean ± SD)	Difference (Mean, 95% CI)	*p*-value (significance: <0.05)
CMS total	50.5 ± 20.8	54.1 ± 20.4	3.6 (− 10.9 to 18.1)	*0.603*
Pain	12.0 ± 3.3	11.2 ± 4.4	−1.1 (− 3.6 to 1.4)	*0.442*
Activity	12.4 ± 5.5	14.4 ± 6.6	2.0 (− 2.0 to 6.0)	*0.357*
Mobility	18.8 ± 9.5	23.1 ± 10.8	4.3 (− 2.5 to 11.1)	*0.231*
Strength	7.0 ± 6.7	5.9 ± 4.6	−1.1 (− 5.5 to 3.3)	*0.503*
Flexion	100.0 ± 44.0	119.2 ± 42.7	19.2 (− 11.3 to 49.7)	*0.188*
Abduction	90.0 ± 39.4	105.0 ± 45.9	15.0 (− 13.4 to 43.4)	*0.317*
SST [%]	74.2 ± 13.2	76.3 ± 19.1	2.1 (− 8.4 to 12.6)	*0.736*
SSV	65.0 ± 23.9	65.1 ± 21.8	0.0 (− 17.2 to 17.2)	*0.995*

*CMS* Constant–Murley Score, *SST* simple shoulder test, *SSV* subjective shoulder value, *95% CI * 95% confidence interval

### Radiological results

Inter-observer reliability between the two raters was excellent (ICC = 0.93) for LHO and good (ICC = 0.85) for LGHO.

**Fig. 2:**
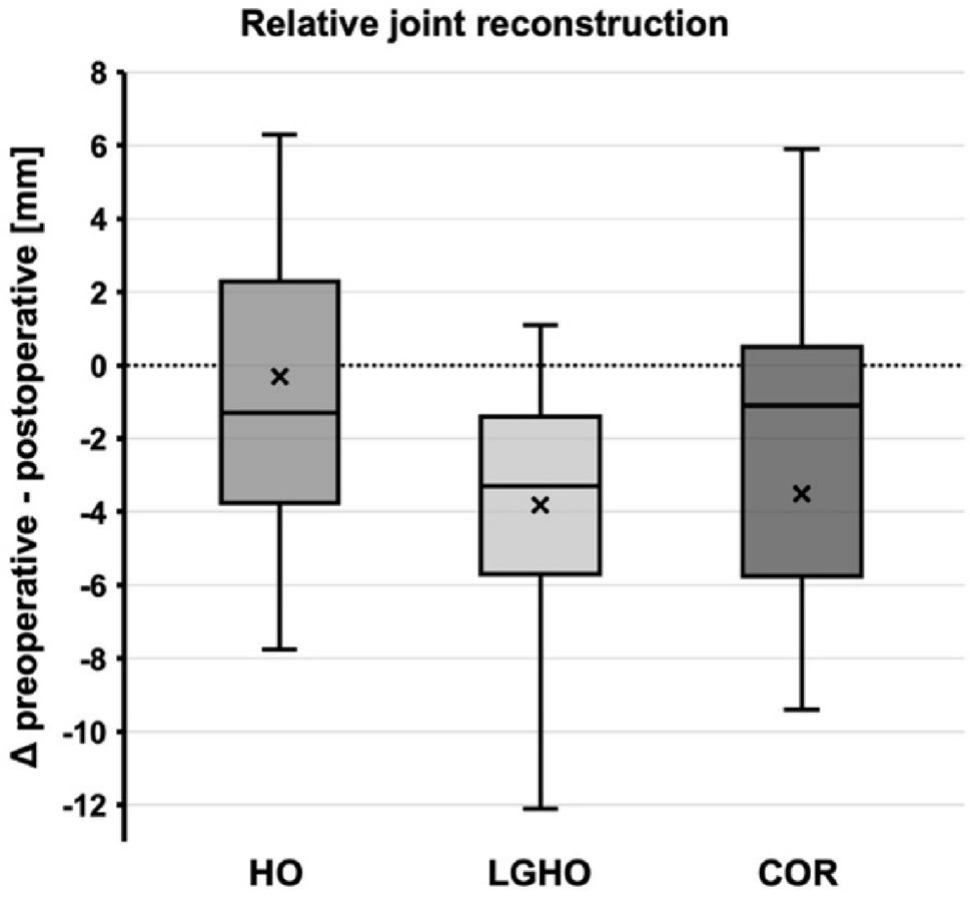
Box-and-Whisker Blot depicting the relative reconstruction of the three offset-parameters from preoperative to the latest follow-up with negative values reflecting a relative under-reconstruction and positive values reflecting relative over-reconstruction: *HO*  Humeral offset, *LGHO* lateral glenohumeral offset, *COR* center of rotation. The box marks the interquartile range, the “x” indicates the mean, the band inside the box indicates the median, whiskers indicating minimum and maximum data

The mean ΔLHO was close to zero (mean ΔLHO = -0.3 mm ± 5.1), LGHO was slightly underreconstructed (ΔLGHO = -6.3 mm ± 12.9) and COR was slightly medialized (ΔCOR = -3.5 mm ± 8.5) (Fig. 2).

Linear regression analysis of the absolute deviations in mm of the reconstruction was performed to evaluate the deviation from postoperative to preoperative offset. The regression revealed an independent negative association of absolute ΔLHO values with clinical outcomes according to CMS. Greater LHO deviation was associated with lower Constant–Murley Score (β = −4.9 points per mm deviation, 95% CI − 9.1 to − 0.8, *p* = 0.024; R² = 0.46) (Table 3).

**Table 3:** Linear regression analysis of offset parameter deviation to preoperative in association with clinical outcome according to the Constant–Murley Score

		Coefficients (95% CI)	Standard Error	*P*-value (significance: <0.05)
abs. ΔCOR-Offset		-1.34 (-4.42–1.74)	1.44	0.367
abs. ΔLHO		-4.94 (-9.11–0.77)	1.94	0.024*
abs. ΔLGHO		2.85 (-0.38–6.07)	1.50	*0.079*
F	3.93			0.032*
R^2^	0.46			
Adjusted R^2^	0.38			

*abs. ΔCOR-Offset*  absolute value of the center-of-rotation offset deviation at the latest follow-up to preoperatively, *abs. ΔLHO* absolute value of the humeral offset deviation at the latest follow-up to preoperatively, *abs. ΔLGHO * absolute value of the lateral glenohumeral offset deviation at the latest follow-up to preoperatively, *F* F statistic of the regression model, *R*^*2*^ R square regression statistic, *R* = 0.68. The asterisk * indicates statistical significance

**Fig. 3:**
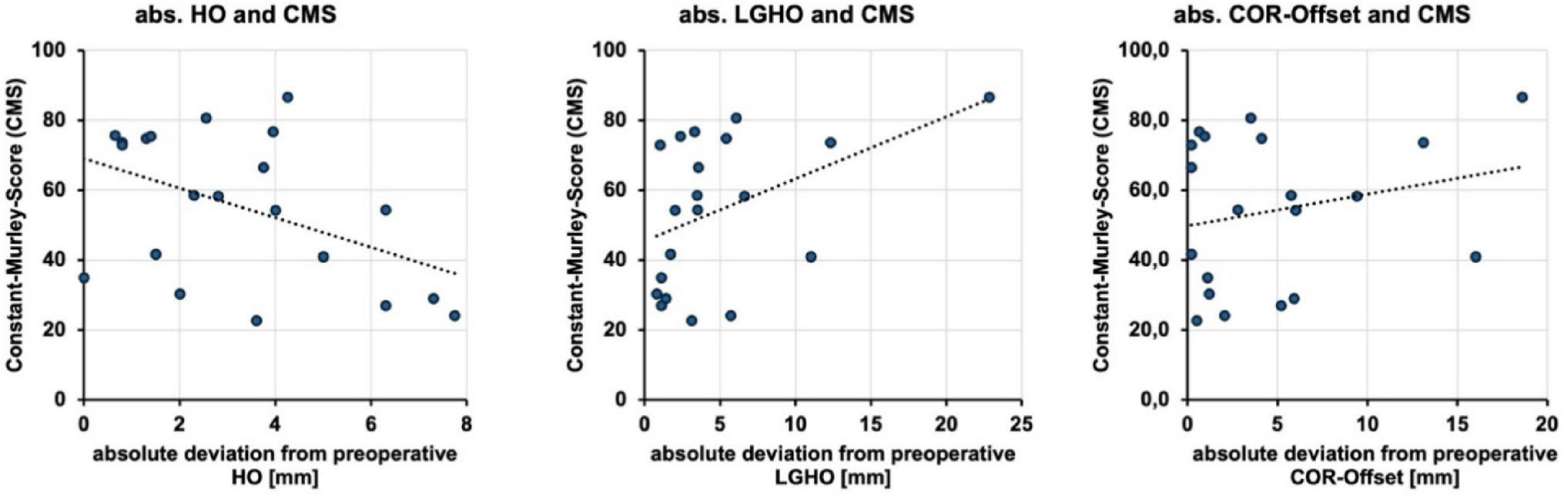
Correlation analysis demonstrating the association between the radiologically measured absolute deviation from preoperative to the latest follow-up for different offset parameters and functional outcome according to the Constant-Murley Score. The dotted line indicates the linear trend line. *HO* humeral offset, *LGHO* lateral glenohumeral offset, *COR* center of rotation

Univariate analysis of the relationship between the absolute deviation of LHO and LGHO to their preoperative values revealed no significant correlation between LHO and CMS (Pearson’s *R* = -0.31, *p* = 0.171), LGHO and CMS (Pearson’s *R* = 0.29, *p* = 0.208) or COR Offset (Pearson’s *R* = 0.23, *p* = 0.322) (Fig. 3). In contrast, the multivariable model above, including LHO, LGHO, and COR offset, identified LHO deviation as significant, likely reflecting shared variance among offset parameters and estimation of the independent contribution of LHO. This is substantiated by a correlation analysis between the three offset parameters (Supplemental Table 2).

## Discussion

The main findings of this study are (1) that both stemmed HA and stemless HA can provide comparable functional outcomes in the long-term with adequate joint reconstruction and (2) that for LHO reconstruction, a greater deviation from the preoperative value was associated with poorer functional outcome. The association of LHO with clinical outcome was independent of the global joint offset parameters LGHO and COR offset, which were highly correlated, reflecting shared variance. A previous study also reported a significant correlation between LHO and Constant–Murley Score in patients undergoing stemmed hemiarthroplasty for proximal humeral fractures [13]. Another study demonstrated worse clinical outcomes in patients with under-reconstruction of the lateral humeral offset following plate osteosynthesis for proximal humeral fractures [1]. However, these studies did not evaluate preoperative offset due to the fracture indication. Nevertheless, their findings support the relevance of accurate LHO reconstruction for postoperative shoulder function. In contrast, changes in glenohumeral offset and COR offset have not been consistently associated with clinical outcome after TSA [3, 5]. Taken together, this evidence aligns with our findings highlighting the importance of minimizing deviations in LHO restoration. Despite the limited patient number, the key strength of the present study lies in the detailed evaluation of individual offset reconstruction using paired pre- and postoperative measurements and their linkage to patient-centered long-term outcome.

Accurate reconstruction of the joint anatomy is recommended to achieve optimal clinical results in TSA [8]. Previous studies assessed joint reconstruction and found that overreconstruction of the lateral humeral offset leads to a temporary deterioration of shoulder function with the first postoperative year [14].

Stemless shoulder arthroplasty offers the theoretical advantage to more freely adjust COR restoration and humeral head reconstruction compared to stemmed implants which set the humeral head component position with modular options. A radiological study comparing different stemless and stemmed humeral prosthetic designs found a more anatomical restoration with a stemless elliptical design [4]. However, Pinto et al. radiologically found similar restoration of the joint anatomy for stemmed and stemless TSA [22]. This aligns with the presented findings of comparable joint restoration across both implant designs. A close-to-anatomic restoration of COR and humeral head positioning was reported for stemless implants [15]. However, these radiological studies did not evaluate clinical outcomes or PROs. The humeral neck cut can pose a surgical challenge, setting up for the implant position of the humeral component: Outliers with failed anatomic restoration of the COR due to inaccurate humeral neck cut were reported to present with lower clinical outcomes, however the number of cases was low in this study [29]. Cox et al. even found worse anatomical humeral reconstruction when using stemless implants compared to short- or standard-stemmed prostheses [7].

Comparative studies of stemmed vs. stemless TSA found similar clinical outcomes and comparable 3D-motion analyzed ROM in a short-term and mid-term follow-up [18, 26]. Uschok et al. observed similar clinical outcomes and comparable coronal plane offset restoration for both stemless and stemmed TSA in a randomized controlled trial with a minimum follow-up of 5 years [28]. A recent comparative study demonstrated a lower number of outliers regarding COR reconstruction in stemless versus short-stemmed TSA [25].

In this data set, LHO was the only offset-parameter showing independent association with functional outcome according to CMS. The strong correlation between COR offset and LGHO indicating shared variance likely reflects their shared geometric basis related to global joint offset restoration. In contrast, LHO primarily reflects humeral-sided reconstruction and soft-tissue tensioning. This may explain why LHO demonstrated an independent association with clinical outcome in the multivariable model.

Although many studies evaluate radiographic joint restoration, few correlate these findings with clinical outcomes.

The investigation of long-term clinical outcomes and associated individual joint offset restoration is the main strength of this study. However, there are limitations that primarily concern the low patient number: The small sample and limited radiographic availability may restrict external validity. Further, the number of patients is too low to allow for statistically robust intergroup comparative analyses. Moreover, radiographic true anteroposterior images of the shoulder can vary in alignment and quality, and no controlled study-specific radiographic protocol was used when taking the images due to the retrospective nature of the analyses. As radiological follow-up was available for only half of the patients with clinical follow-up, an intergroup comparison was performed between patients with complete clinical but incomplete radiological follow-up and those with complete datasets. This analysis revealed no significant differences between the groups, except for a longer mean follow-up duration in patients with incomplete radiological follow-up. This is likely due to the earlier operative dates in this subgroup, as patients treated in earlier years were less likely to undergo digital, calibrated preoperative radiographic assessment.

Only 25 of 48 patients had complete paired radiographs suitable for offset analysis, which limited statistical precision and may introduce selection bias. Furthermore, with three predictors and a small sample (~ 8 observations per predictor), regression coefficients and model fit estimates may be optimistic and susceptible to overfitting. Consequently, these findings should be interpreted as exploratory and require validation in larger cohorts.

Despite its limitations the study can provide valuable and unique data on offset reconstruction in stemmed and stemless HA and their association with long-term clinical outcomes.

## Conclusion

Both stemmed and stemless HA can achieve acceptable to good long-term clinical outcomes with satisfactory joint geometry restoration. Higher deviation in LHO reconstruction is associated with worse long-term outcomes in this selected patient cohort.

## Supplementary Information

Below is the link to the electronic supplementary material.

Supplementary Material 1

## Data Availability

No datasets were generated or analysed during the current study.
