# Supplementary material for: Offset reconstruction in stemless and stemmed shoulder hemiarthroplasty: influence on long-term outcomes
Source: Arch Orthop Trauma Surg. 2026 Jun 3;146(1):207. doi: 10.1007/s00402-026-06314-3 (PMC13234034; doi:10.1007/s00402-026-06314-3)
Supplement: Supplementary file 1 — Supplementary Material 1 [file 402_2026_6314_MOESM1_ESM.docx]

| **Outcome** | **RFU complete (Mean ± SD)** | **RFU incomplete (Mean ± SD)** | **p-value (t-test)** |
| --- | --- | --- | --- |
| **Total number** | 25 | 23 |  |
| **Age** | 56 ± 11.4 | 54.1 ± 9.1 | 0.527 |
| **Follow-up (months)** | 185.6 ± 28.9 | 214.6 ± 22.7 | 0.0003 |
| **CS Total** | 53.7 ± 20.7 | 52.7 ± 20.4 | 0.875 |
| SST [%] | 74.9 ± 17.36 | 76.9 ± 18.7 | 0.712 |
| **SSV** | 61.7 ± 22.5 | 66.8 ± 23.3 | 0.450 |

Supplemental Table 1: Intergroup comparison of patients with radiological follow-up (RFU) complete vs. incomplete.

| Parameter | **COR** | **LHO** | **LGHO** |
| --- | --- | --- | --- |
| **COR** | 1.00 | 0.11 | **0.86** |
| **LHO** | 0.11 | 1.00 | 0.07 |
| **LGHO** | **0.86** | 0.07 | 1.00 |

Supplemental Table 2. Correlation analysis between offset deviation parameters. Pairwise associations between absolute deviations of COR offset, lateral humeral offset (LHO), and lateral glenohumeral offset (LGHO) were assessed using Pearson’s correlation coefficients. Correlation values represent the strength and direction of linear relationships between parameters.
